# Attitudes towards smoking and COVID-19, and changes in smoking behaviors before and after the outbreak of COVID-19: A nationwide cross-sectional survey study in China

**DOI:** 10.18332/tid/144242

**Published:** 2022-02-17

**Authors:** Yanhui Liao, Jinsong Tang, Anne C.K. Quah, Geoffrey T. Fong, Ann McNeill

**Affiliations:** 1 Department of Psychiatry, Sir Run Run Shaw Hospital, Zhejiang University School of Medicine, Hangzhou, China; 2 Department of Addictions, Institute of Psychiatry, Psychology and Neuroscience, King's College London, London, United Kingdom; 3 Department of Psychology, University of Waterloo, Waterloo, Canada; 4 School of Public Health Sciences, University of Waterloo, Waterloo, Canada; 5 Ontario Institute for Cancer Research, Toronto, Canada

**Keywords:** smokers, non-smokers, smoking behaviors, attitudes, COVID-19

## Abstract

**INTRODUCTION:**

China has more than 300 million current smokers. There is a controversy over smokers’ risk of COVID-19 infection. Smoking is a risk factor for COVID-19 disease progression, and the outbreak of COVID-19 may change people’s smoking behaviors. This study assessed people’s attitudes towards ‘smoking and COVID-19’ and changes of smoking behaviors before and after the outbreak of COVID-19.

**METHODS:**

A cross-sectional web survey of 11009 adults in China was conducted between 7 May and 3 August 2020. Attitudes towards ‘smoking and COVID-19’ were compared among non-smokers (n=8837), ex-smokers (n=399) and current smokers (n=1773), and changes in smoking behaviors before and after the outbreak of COVID-19 were assessed among current smokers.

**RESULTS:**

Fewer current smokers (26.2%) agreed with the statement that ‘Current smokers are more likely than ex-smokers or non-smokers to contract COVID-19’ compared with non-smokers (53%) or ex-smokers (41.4%); fewer current smokers (55.9%) agreed with the statement ‘If contracted, current smokers are more likely than ex-smokers or non-smokers to risk disease progression’ compared with non-smokers (75.5%) or ex-smokers (68.7%). There were no changes in cigarettes smoked per day (mean ± SD: 13.3 ± 9.55 vs 13.4 ± 9.69, p=0.414), percentage of daily smokers (70.8% vs 71.1%, p=0.882) and percentage of smokers with motivation to quit (intend to quit within the next 6 months, 9.4% vs 10.9%, p=0.148) before and after the outbreak of COVID-19.

**CONCLUSIONS:**

The survey found that fewer current smokers agreed that smoking is a risk-factor for COVID-19 compared with non-smokers or ex-smokers. Among current smokers, there were no changes in their cigarette consumption and motivation to quit before and after the outbreak of COVID-19. More efforts are needed to educate smokers about the health risks of smoking, as well as efforts to promote their motivation to quit.

## INTRODUCTION

Approximately one-third of the world’s smokers (>300 million) live in China and they consume more than 40% of the world’s total cigarettes^1,2^. A large survey of adults in the UK found that prevalence of confirmed COVID-19 was higher among current smokers (0.56%) than never smokers (0.26%) and ex-smokers (0.19%)^3^. Research indicates that smoking is a risk factor for COVID-19 disease progression^4-6^, especially among younger adults^7^. However, evidence also showed a low prevalence of current smoking among hospitalized patients with COVID-19, and current smokers were more likely to have an adverse outcome compared with non-current smokers but less likely compared with ex-smokers^8^.

The outbreak of COVID-19 may change people’s smoking behaviors. A survey study in England examined the changes in smoking behaviors following the COVID-19 lockdown and found that smokers are more likely than before the lockdown to report trying to quit smoking, and rates of smoking cessation are higher^9^. However, these changes remain unknown among people from China.

The primary objective of this study is to assess the attitudes of current smokers, ex-smokers and non-smokers towards ‘smoking and COVID-19’ and changes of smoking behaviors among Chinese before and after the outbreak of COVID-19. Considering people received a great deal of information about COVID-19, as well as well-documented knowledge about the association between smoking and health risks, we hypothesized that most people would agree that smoking was a risk-factor for COVID-19; and that compared with before the outbreak of COVID-19, Chinese smokers might smoke fewer cigarettes per day and have stronger motivation to quit smoking at present.

## METHODS

### Participants and design

The sample included 11009 participants from China. Participants were: aged ≥18 years; willing to participant, being able to read Chinese; and being able to complete an online survey.

All eligible participants were invited to complete sociodemographic information and attitudes towards ‘smoking and COVID-19’.

The web survey was conducted from 7 May to 3 August 2020. Informed consent was obtained from each eligible participant before completing a 10–30 min demographic questionnaire and measures of addictive behaviors (data on other addictive behaviors were reported elsewhere^10^).

The web survey was performed using a professional online survey service Questionnaire Star (https://www.wjx.cn), released nationwide through social media (such as WeChat, Weibo, QQ). A previous study has indicated the reliability and validity of online surveys for assessing smoking behaviors and thoughts^11^. The survey allowed only one response per phone or computer. Considering that respondents could share the same internet and same IP address with their families, this study did not limit the number of questionnaires from the same IP address. However, the data had no multiple entries with the same IP address.

### Participation

This study did not involve patients. Participants were not involved in the design of the research. This study was in compliance with the principles included in the Declaration of Helsinki. The research protocol was approved by The Ethics Committee of Sir Run Run Shaw Hospital, an affiliate of Zhejiang University, Medical College (No. 2020-0505-33). Participants were informed about the purpose, assessments, potential risks and benefits of the study before consenting. Digital consent form was provided to each participant. At the bottom of the consent form, they could choose to ‘agree to participate’ or ‘disagree to participant’. No personal information was collected, and all information from the data cannot be linked back to the participants.

### Measures

Smoking status was based on the following definitions:

Current smokers – those who have smoked 100 cigarettes in their lifetime and currently smoke daily (‘everyday’ smoker) or on some days (‘somedays’ smoker);

Ex-smokers – those who have smoked at least 100 cigarettes in their lifetime but had quit smoking in the last 28 days at the time of the survey; and

Never smokers or non-smokers – those who had smoked less than 100 cigarettes in their lifetime, and currently not smoking.

Current smokers and ex-smokers completed questions about their smoking history and quitting information. Current smokers also completed questions about smoking behaviors (quantity and frequency) and motivation to quit before (one month before December 2019) and after the outbreak of COVID-19 (within one month of the survey period 7 May to 3 August 2020), as well as nicotine dependence (Fagerström test for nicotine dependence, FTND) at the time of the survey. Attitudes towards ‘smoking and COVID-19’ were asked by the following two questions: ‘Are current smokers more likely than ex-smokers or non-smokers to contract coronavirus disease 2019 (COVID-19)?’ and ‘If contracted, are current smokers more likely than ex-smokers or non-smokers to risk disease progression?’. Each question used a 5-point Likert scale: ‘strongly agree’, ‘somewhat agree’, ‘neither agree nor disagree’, ‘somewhat disagree’, and ‘strongly disagree’.

### Data analysis

A user-specified Excel file was downloaded from the database. Statistical analysis was performed using SPSS (Version 22.0). Descriptive statistics, chi-squared (χ^2^) test and dependent t-tests were applied to measure outcomes. Statistical significance was assumed for two-sided p<0.05.

## RESULTS

### Sociodemographic information

Among the 11009 participants, 42.8% were males, 55.2% were aged ≤34 years, 96.1% received tertiary education, 96.3% were employed, 59.5% were married, 94.9% were from urban areas, 80.3% were non-smokers, 16.1% were current smokers, 3.6% were ex-smokers, and 6.26% (n=136, among current smokers and ex-smokers) had made a quit attempt during the outbreak of COVID-19. The average age (mean ± SD) was 34.9 ± 10.13 in males and 32.3 ± 9.82 in females (p<0.001).

### Sociodemographic information by gender

Among males, 57.6% were non-smokers, 34.8% were current smokers, and 7.6% were ex-smokers; among females, 97.3% were non-smokers, 2.1% were current smokers, and 0.6% were ex-smokers.

### Sociodemographic information by smoking status

Non-smokers (n=8837) were 30.7% male, 59.2% aged ≤34 years, 93.3% received tertiary education, 96.6% were employed, 56.4% were married, and 94.7% were from urban areas. Current smokers (n=1773) were 92.5% male, 41.2% aged ≤34 years, 84.8% received tertiary education, 95.7% were employed, 70.4% were married, and 96% were from urban areas. Ex-smokers (n=399) were 90.2% male, 28.1% aged ≤34 years, 83.2% received tertiary education, 91.7% were employed, 80.2% were married, and 96.5% were from urban areas.

### Attitudes towards ‘smoking and COVID-19’

**Figure 1:**
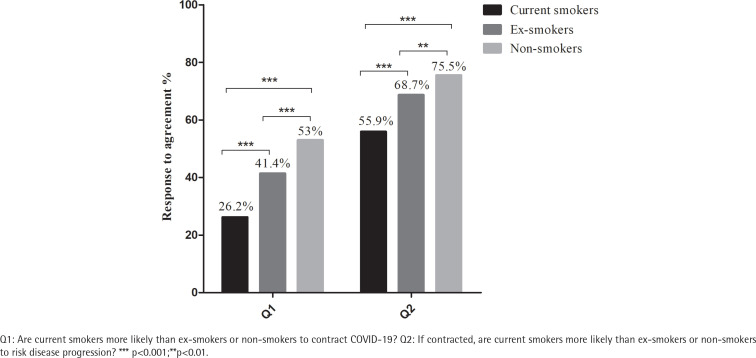
Percentage of agreement to ‘smoking is a risk-factor for COVID-19’ among current smokers, ex-smokers, and non-smokers

Current smokers, ex-smokers and non-smokers agreeing (‘strongly agree’ and ‘somewhat agree’) that ‘smoking is a risk-factor for COVID-19’ are shown in Figure 1. Fewer current smokers (26.2%) agreed that smoking was a risk-factor for COVID-19 infection compared with non-smokers (53%) or ex-smokers (41.4%) (all p<0.001). Fewer current smokers (55.9%) agreed that smoking was associated with worse outcomes of COVID-19 compared with non-smokers (75.7%) or ex-smokers (68.7%) (all p<0.001). Furthermore, fewer ex-smokers agreed that smoking was a risk-factor for COVID-19 infection (p<0.001), and that smoking was associated with worse outcomes of COVID-19 (p=0.002) compared with non-smokers.

### Smoking behaviors before and after the outbreak of COVID-19

Comparing the smoking behaviors of current smokers before and after the outbreak of COVID-19, there were no changes in cigarette consumption (number of cigarettes smoked per day 13.3 ± 9.55 vs 13.4 ± 9.69, p=0.414), percentage of daily smokers (70.8% vs 71.1%, p=0.882) and percentage of smokers with motivation to quit (within the next 6 months, 9.4% vs 10.9%, p=0.148). Almost half of (45.2%) current smokers had no previous quit attempts, but 96% of ex-smokers had made multiple previous quit attempts. Nicotine dependence at the time of the survey was reported by current smokers as follow: 45.9% very low/no dependence (FTND score: <4), 43.3% low to medium dependence (FTND score: 4–6), and 10.8% high dependence (FTND score: >6).

## DISCUSSION

Compared with non-smokers, fewer current smokers agreed that smoking was a risk-factor for COVID-19. The outbreak of COVID-19 did not change their cigarette comsumption and did not motivate them to quit smoking.

More than half of non-smokers agreed with the statement ‘Current smokers are more likely than ex-smokers or non-smokers to contract coronavirus disease 2019 (COVID-19)’, but only about a quarter of current smokers agreed with it. Even with evidence showing that smoking has been associated with COVID-19 disease progression^5^, only around half of current smokers agreed with the statement ‘If contracted, current smokers are more likely than ex-smokers or non-smokers to risk disease progression’, which was significantly lower than that among non-smokers, indicating the lack of knowledge about the harms of smoking among current smokers^12^.

This study also found there were no significant changes in smoking behaviors before and after the outbreak of COVID-19. Smokers smoked almost the same amount of cigarettes per day, and about 70% were daily smokers. Furthermore, there were also no significant changes in motivation to quit, with very few (about 10%) current smokers considering quiting smoking within the next 6 months. However, research from England^9^ and Hong Kong^13^ found that the COVID-19 lockdown was associated with increased motivation to quit and cessation. Furthermore, a Dutch online survey found that smokers smoked less (14.1%) or more (18.9%) cigarettes due to the COVID-19 pandemic^14^. Although no statistically significant differences were observed in motivation to quit before and after the outbreak of COVID-19, slightly more current smokers (1.5%) were motivated to quit after the outbreak of COVID-19. Promoting current smokers to quit can minimize the risks associated with the current coronavirus pandemic. Implementing stronger tobacco control policies in China during the outbreak of COVID-19 may greatly encourage smokers to quit.

### Limitations

The study had several limitations. First, the sample was not representative of the adult population of China. According to China’s National Bureau of Statistics (http://www.stats.gov.cn/tjsj/ndsj/2020/indexch.htm), 48.91% of the Chinese population are female, and urban population accounts for 60.6% of the total population and the prevalence of smoking in the current study is lower (16.1%) than that of the general population (26.6%) reported by The Global Adult Tobacco Survey (GATS) China 2018 (https://www.tobaccofreekids.org/assets/global/pdfs/en/GATS_China_2018_FactSheet.pdf). Second, in addition to selection bias, recall bias would have occurred due to the retrospective nature of the study. Third, changes in smoking behavior were not examined among former smokers. Fourth, this survey assessed quit attemps during the outbreak of COVID-19, but did not assess whether current and ex-smokers had attempted to quit or stopped smoking due to COVID-19. If they had tried and failed in their quit attempts, then this could have impacted their attitudes. Fifth, data were collected relatively early in the pandemic, participants’ attitudes and smoking behaviors may have changed since then.

## CONCLUSIONS

This study found that, unlike non-smokers and ex-smokers, many current smokers did not think that smoking is a risk-factor for COVID-19, and smokers from this sample neither reduced their cigarette consumption nor increased their motivation to quit smoking during the outbreak of COVID-19. More efforts are needed to educate smokers about the health risks of smoking, as well as efforts to promote their motivation to quit smoking.

## Data Availability

The data supporting this research are available from the authors on reasonable request.
